# NF‐κB p65 promotes ovarian cancer cell proliferation and migration via regulating mortalin

**DOI:** 10.1111/jcmm.14325

**Published:** 2019-04-14

**Authors:** Shan Li, Mengyuan Lv, Shi Qiu, Jiaqi Meng, Wen Liu, Ji Zuo, Ling Yang

**Affiliations:** ^1^ Department of Cellular and Genetic Medicine, School of Basic Medical Sciences Fudan University Shanghai China

**Keywords:** migration, mortalin, NF‐κB, ovarian cancer, proliferation

## Abstract

Previous studies show that mortalin, a HSP70 family member, contributes to the development and progression of ovarian cancer. However, details of the transcriptional regulation of mortalin remain unknown. We aimed to determine whether NF‐κB p65 participates in the regulation of mortalin expression in ovarian cancer cells and to elucidate the underlying mechanism. Chromatin immunoprecipitation and luciferase reporter assay were used to identify mortalin gene sequences, to which NF‐κB p65 binds. Results indicated that NF‐κB p65 binds to the mortalin promoter at a site with the sequence ‘CGGGGTTTCA’. Using lentiviral pLVX‐NF‐κB‐puro and Lentivirus‐delivered NF‐κB short hairpin RNA (shRNA), we created ovarian cancer cell lines in which NF‐κB p65 was stably up‐regulated and down‐regulated. Using these cells, we found that downregulation of NF‐κB p65 inhibits the growth and migration of ovarian cancer cells. Further experimental evidence indicated that downregulation of NF‐κB p65 reduced mortalin, and upregulation of mortalin rescued the proliferation and migration of ovarian cancer cells reduced by NF‐κB p65 knockdown. In conclusion, NF‐κB p65 binds to the mortalin promoter and promotes ovarian cancer cells proliferation and migration via regulating mortalin.

## 1 INTRODUCTION

Ovarian cancer is one of the malignant tumours in women. As there are no reliable early symptoms and signs of ovarian cancer, its early detection is difficult. This feature, accompanied with the poor efficacy of treatment of advanced stages, results in a high mortality rate,^1^ making it a serious concern for women's health.^2^, ^3^

Mortalin, also known as GRP75/HSPA9, is a mitochondrial molecular chaperone of HSP70 family. It participates in many cellular activities, including protein folding and transport, maintenance of mitochondrial steady state, material synthesis, cell apoptosis, aging and intracellular signalling pathways.^4^, ^5^ Mortalin is elevated in most malignant tumours such as breast,^6^ liver,^7^ lung^8^ and gastric cancers,^9^ and its overexpression can promote tumour cell migration and invasion.^10^ Our previous studies have shown that mortalin affects proliferation, migration and drug sensitivity of ovarian cancer cells through various signalling pathways such as P38/MAPK, PI3K/AKT and MAPK/ERK.^11^ Our other studies suggest that mortalin is a potential drug target that could be used to treat disease including tumours.^12^ However, the mechanism underlying mortalin regulation remains unclear.

Transcription factors are proteins that regulate the transcription of genes by binding to the gene promoter regions. They play an important role in tumorigenesis, tumour development, infiltration and metastasis, making them potential targets for anti‐tumour drugs.^13^, ^14^, ^15^, ^16^ Nuclear transcription factor kappa B (NF‐κB) is a transcription factor that was first discovered in B cells. It specifically binds to the enhancer κB sequence of the immunoglobulin kappa light chain gene and is involved in the body's inflammatory and immune responses.^17^ NF‐κB is present in various tissues and cells and plays critical roles in cell proliferation and cell death. It also regulates cell differentiation, inflammation and other pathological processes.^18^, ^19^ NF‐κB has been implicated in various diseases such as allergies,^20^ neurodegenerative diseases,^21^, ^22^ ophthalmic diseases^23^, ^24^ and cancer.^25^, ^26^, ^27^, ^28^ There are five members of the NF‐κB family, p65 (RelA), RelB, c‐Rel, p105/p50 (NF‐κB1) and p100/52 (NF‐κB2).^29^ NF‐κB p65 subunit is considered as the most potent transcriptional activator of the family.^30^

This study was prompted by our earlier study that used bioinformatics techniques to predict and determine the potential transcription factors of mortalin. Results showed NF‐κB p65 and MZF‐1 were the potential transcription factors of mortalin (Supporting Information Table S1). We selected NF‐κB p65 for further study and assessed whether it participates in the regulation of mortalin expression in ovarian cancer cells.

## 2 MATERIALS AND METHODS

### 2.1 Cell lines and cell culture

The cisplatin‐resistant human ovarian cancer cell line A2780CP and the cisplatin‐sensitive human ovarian cancer cell line A2780S, 293FT and 293T cells were cultured in Dulbecco's modified Eagle medium (DMEM, Gibco, NY, USA) supplemented with 10% foetal bovine serum (FBS, HyClone, GE, USA) at 37°C under humidified atmosphere containing 5% CO_2_.

### 2.2 Plasmid constructs, transfection and infection

NF‐κB p65 overexpression and interferon expression lentiviral vectors were constructed in the pLVX‐IRES‐Puro and pLVX‐shRNA1 vectors by inserting the NF‐κB p65 PCR fragment and shRNA, respectively, into the BamHI/EcoRI sites of the vectors. Empty pLVX‐IRES‐Puro and pLVX‐shRNA1 were used as controls. Mortalin‐overexpression lentiviruses (mortalin‐pLVX‐AcGFP) was saved in our laboratory.

A2780CP and A2780S were transfected with NF‐κB p65 overexpression and interferon expression lentiviral vectors to generate stable cell lines. Additionally, we introduced the mortalin‐overexpression vector into the NF‐κB p65 down‐regulated A2780CP cells. After transfection, cells were cultured for 72 hours and then screened after approximately 1 week using 2 µg/mL puromycin to obtain the stable cell lines.

293T cells were cultured in DMEM containing 10% FBS, at 37°C and in 5% CO_2_. Logarithmic phase 293T cells were trypsinized, seeded in 10 cm culture dishes and used for transfection once they reached 60%‐80% confluence. Three systems of plasmid, including the constructed vectors psPAX2 and PMD2G were used to transfect 293T cells. HilyMax (Dojindo, Kumamoto, Japan) was used to transfect the cells following the manufacturer's instructions. The 48 and 72 hours supernatants were collected and used for further infections.

### 2.3 Real‐time quantitative PCR

Total RNA was extracted from cells using the RZ reagent according to the instructions in the RNAsimple total RNA Kit (TIANGEN, Beijing, China). First‐stand cDNA was synthesised using the HiScript 1st Strand cDNA Synthesis Kit (Vazyme, Nanjing, China). Real‐time quantitative PCR (RT‐qPCR) was performed with the FastStart Universal SYBR Green Master (ROX) (Roche, Basel, Switzerland) and the Light Cycler Nano real‐time fluorescence quantification PCR system (Roche, USA). Relative expression of genes was calculated using the 2^−△△^Ct method. The primers used were:

β‐actin‐FW 5'‐ TTGCCATCAATGACCCCTTCA −3’;

β‐actin‐RV: 5'‐ CGCCCCACTTGATTTTGGA −3';

NF‐κB p65‐Fw: 5'‐ CGCGGATCCGCCACCATGGACGAACTG‐3';

NF‐κB p65‐Rv: 5'‐ CCGCTCGAGTTAGGAGCTGATCTG‐3';

Mortalin ‐ FW: 5'‐TCTGCTGTAAAGGCCACAAC‐3';

Mortalin ‐ RV: 5'‐ CAGGAGTTGGTAGTACCCAAATC −3';

NF‐κB p65‐chip‐Fw: 5'‐ TCAGCGGAAGAGCGG‐3'.

NF‐κB p65‐chip ‐Rv: 5'‐AGGAGTACGAGGCAG‐3'.

### 2.4 Western blotting

Radioimmunoprecipitation (RIPA) lysis solution supplemented with 1mM phenylmeth‐ylsulfonyl fluoride (PMSF) and 1% protease inhibitor cocktail was used to lyse cells and obtain total protein. A BCA Protein Assay Kit (Thermo Fisher Scientific, Waltham, MA, USA) was used to determine the protein concentration. Proteins were separated by a sodium dodecyl sulphate polyacrylamide gel and then transferred to a protein‐blotted polyvinylidene membrane (PVDF, GS0914; Millipore, Billerica, MA, USA). The membrane was blocked with 5% skimmed milk for 2 hours and then incubated with anti‐NF‐κB p65 (Cell Signaling Technology, USA), anti‐mortalin (Cell Signaling Technology, MA, USA) and anti‐β‐actin (Sigma‐Aldrich, MO, USA) primary antibodies at 4°C overnight. Membranes were then incubated with secondary antibody for 2 hours at room temperature. Proteins were then detected using Immobilon Western chemiluminescent HRP substrate (Bio‐Rad, Hercules, CA, USA) and a Gel Doc XR System (Bio‐Rad). Results were analysed with Image J software (NIH, Bethesda, MD, USA).

### 2.5 Immunofluorescence

To detect NF‐κB p65 and mortalin expression and their cell localisation in cell lines. Cells were seeded on glass slides coated by poly‐l‐lysine (Sigma‐Aldrich, MO, USA), 4% paraformaldehyde fixed 15 minutes and permeabilised with 0.5% TritonX‐100 for 10 minutes. Then cells were blocked with 5% FBS for 30 minutes, incubated with anti‐NF‐κB p65 (Cell Signaling Technology, MA, USA) or anti‐mortalin (Cell Signaling Technology, MA, USA) primary antibodies at 4°C overnight. After three times washed with 0.1M PBS, cells were incubated with fluorescent secondary antibody. DAPI or Hochest was used to stain nucleus. Fluorescence microscope was then applied to capture images.

### 2.6 Cell viability assay

Cell viability was assessed using a Cell Counting Kit‐8 (CCK‐8, Dojindo, Kumamoto, Japan). Cells were seeded in 96‐well plates at a density of 5000 cells/well and cultured at 37°C for 24 hours. After incubation with CCK‐8 reagent for 2 hours at 37°C, the optical density was measured at 450 nm by using a Multiskan MK3 microplate reader (Thermo Fisher Scientific, MA, USA).

### 2.7 Colony formation assay

Cells were seeded into 6 cm dishes at 1000 cells per dish. After incubation for 2 weeks, cells were washed with PBS, fixed with methanol for 10 minutes, stained with 10% Giemsa solution for 15 minutes, washed with running water and dried before being photographed. Visible colonies were counted.

### 2.8 Cell migration assay

Cell migration was detected by wound healing assay. Cells were seeded into a 6‐well plate at the density of 2 × 10^6^ cells per well. When cells reached full confluence, a wound was scratched across the middle of each well using a micropipette tip. Cells were washed twice with PBS and cultured with fresh DMEM medium containing mitomycin C (10 ug/mL; Sigma‐Aldrich, MO, USA). Cells were photographed at the beginning (0 hour) using a DM2500 fluorescence microscope (Leica, Germany). And after the cells incubated for 24 hours, the same fields were photographed again (24 hours). ImageJ software (NIH, USA) was used to evaluate the average extent of wound closure by measuring the width of the wound.

### 2.9 Chromatin immunoprecipitation assay

The chromatin immunoprecipitation (CHIP) assay was performed according to the instructions provided with the CHIP assay kit (Sigma‐Aldrich, MO, USA). Briefly, 10% of the chromatin was saved to act as the input control and remainder diluted in CHIP dilution buffer. The diluted chromatin was incubated with 5 μL anti‐NF‐κB p65 antibody or normal immunoglobulin G (IgG). Immunoprecipitated DNA was analysed using PCR and RT‐qPCR.

### 2.10 Luciferase reporter assay

NF‐κB p65 possible binding sequence of mortalin promoter (TCAGTAGAGACGGGGTTTCACCGTGTTAGC) was cloned into the pGL4.10‐luc2 vectors to generate luciferase reporter plasmid (pGL4.10‐proMortalin‐luc). HEK‐293FT cells, A2780CP cells and A2780S cells were seeded into 24‐well plates. Once reaching a confluence of 60%, the cells were transfected with different plasmids (100 ng of pGL4.10‐basic‐luc or pGL4.10‐proMortalin‐luc and 5 ng of pRL‐TK vectors; 100 ng of pGL4.10‐proMortalin‐luc, 100ng NF‐κB p65 overexpressing vectors and 5 ng of pRL‐TK vectors). The luciferase activities were detected 36 hours after transfection using the Dual‐Luciferase Reporter Assay System (Beyotime, Shanghai, China) according to the manufacturer's protocol. Firefly luciferase activities were normalised to renilla luciferase values, and expressed as relative luciferase units.

### 2.11 Statistical analysis

SPSS 12.0 statistical software (IBM) was used to analyse the experimental data. Independent sample t‐tests and single factor analysis of variance (One ‐ way ANOVA) were used for statistical analysis. *P* < 0.05 was considered statistically significant.

## 3 RESULTS

### 3.1 NF‐κB binds to the mortalin promoter

**Figure 1:**
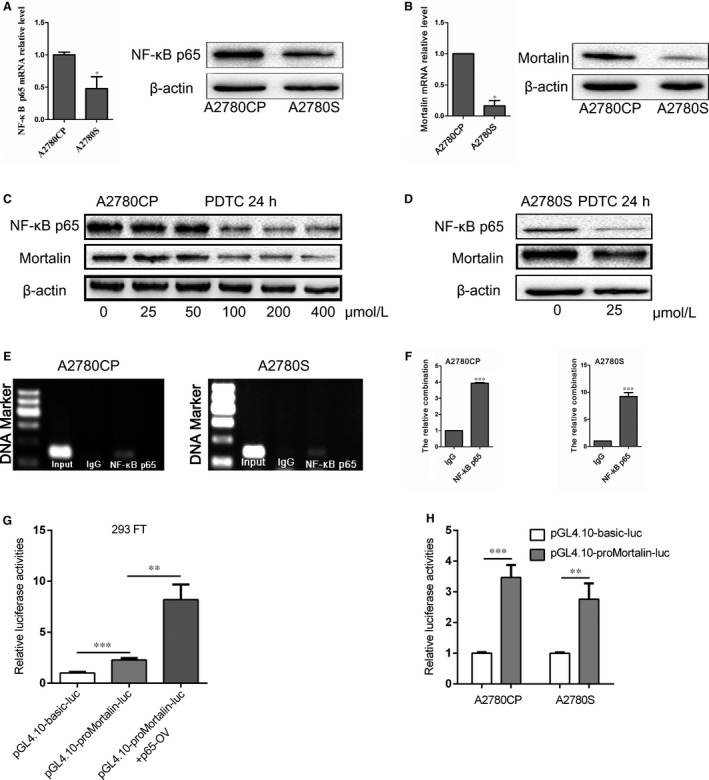
NF‐κB p65 binds to the mortalin promoter. (A) Real‐time quantitative PCR (RT‐qPCR) and Western blotting analysis of NF‐κB p65 expression in A2780CP and A2780S cells. (B) The mRNA and protein expression of mortalin in A2780CP and A2780S cells were evaluated by RT‐qPCR and Western blotting. (C) After treated with different concentrations of NF‐κB p65 inhibitor PDTC for 24h, the NF‐κB p65 and mortalin protein expression in A2780CP cells were measured by Western blotting. (D) After treated with 25 μmol L^−1^ PDTC for 24h, the NF‐κB p65 and mortalin protein expression in A2780S cells were measured by Western blotting. (E) PCR following CHIP showed that NF‐κB p65 binds to the promoter region of mortalin. (F) CHIP combined with RT‐qPCR results showed that anti‐NF‐κB p65 group was higher than IgG group. The m1 primers were used. (G) For the luciferase assay, HEK293FT cells were transfected with the pGL4.10‐proMortalin‐luc vector and NF‐κB p65 overexpressing vector to access the p65 binds to mortalin promoter. (H) Detection of NF‐κB p65 binding site on the mortalin promoter by luciferase reporter assay in A2780CP and A2780S cells. *P < 0.05, **P < 0.01, ***P < 0.001

Real‐time quantitative PCR and Western blotting were used to detect the expression of NF‐κB p65 and mortalin in ovarian cancer cells. The mRNA and protein expression of NF‐κB p65 in the cisplatin‐resistant cell line A2780CP was higher than in cisplatin‐sensitive cell line A2780S (Figure 1A). Concurrently, the mRNA and protein expression of mortalin in A2780CP cells were also higher than in A2780S cells (Figure 1B). Pyrrolidine dithiocarbamate (PDTC) is a specific inhibitor of NF‐κB that can inhibit the expression of NF‐κB p65 subunit and the degradation of IκB, and reduce the nuclear translocation of NF‐κB.^31^ Therefore we incubated A2780CP and A2780S cells with different concentrations of PDTC for 24 hours and then examined NF‐κB p65 and mortalin expression by Western blotting. After treatment of PDTC, the protein levels of NF‐κB p65 and mortalin in A2780CP cells were both reduced in a dose‐dependent manner (Figure 1C). Similarly, the expression of NF‐κB p65 and mortalin in A2780S cells decreased after treatment with PDTC (25 μmol L^−1^) for 24 hours (Figure 1D). This indicated that the inhibition of NF‐κB could reduce the protein expression of mortalin in ovarian cancer cells.

The relationship between NF‐κB p65 and mortalin was confirmed using a CHIP assay coupled with RT‐qPCR. Four primer pairs were designed (Supporting Information Table S2). RT‐qPCR results showed that there were no significant differences between anti‐NF‐κB p65 and the control IgG, if the m2, m3, m4 primers were used (Supporting Information Figure S1). The results of PCR, in which the m1 primers were used, showed the anti‐NF‐κB p65 and input group shared a similar band separation both in A2780CP and A2780S cells (Figure 1E). RT‐qPCR showed similar results that the value of anti‐NF‐κB p65 group was higher than that of IgG group (*P* < 0.05) (Figure 1F). The CHIP results suggest that NF‐κB p65 could combine with mortalin promoter, and the possible binding site is ‘CGGGGTTTCA’.

We next assessed whether NF‐κB p65 regulated mortalin transcription in this binding site using a luciferase reporter assay. NF‐κB p65 possible binding sites of mortalin promoter (CGGGGTTTCA) were cloned into the luciferase reporter plasmid (pGL4.10‐proMortalin‐luc). In 293FT cells, the relative luciferase activity was higher in pGL4.10‐proMortalin‐luc group compared with the pGL4.10‐basic‐luc group (Figure 1G). And transfection of NF‐κB p65 overexpression vectors increased the luciferase activities of pGL4.10‐proMortalin‐luc group. We confirmed this in the ovarian cancer cells; the relative luciferase activity of pGL4.10‐proMortalin‐luc cells was higher than that of pGL4.10‐basic‐luc cells (Figure 1H). All these indicated that NF‐κB p65 could bind to the sequence ‘CGGGGTTTCA’ in mortalin promoter.

### 3.2 NF‐κB p65 regulates the expression of mortalin

**Figure 2:**
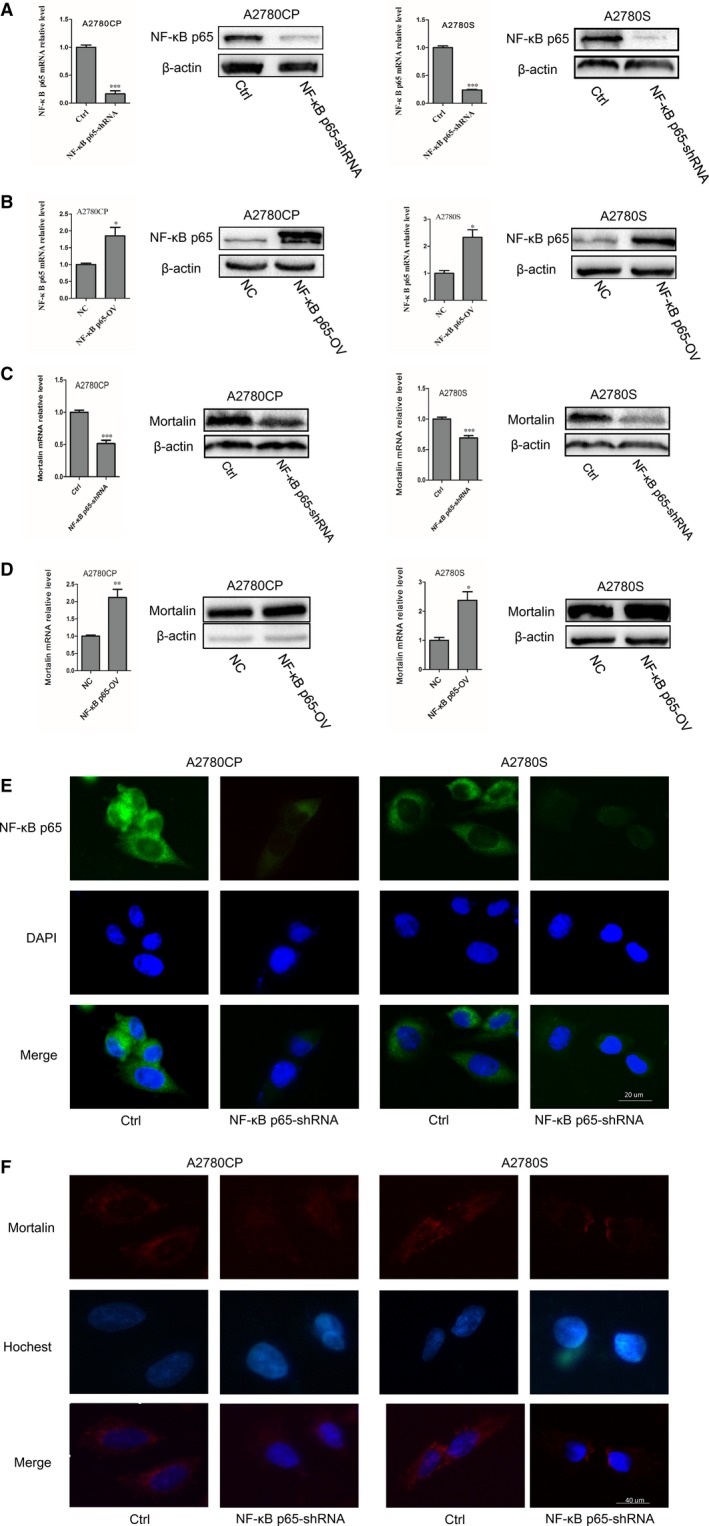
NF‐κB p65 regulates the expression of mortalin. (A) NF‐κB p65 mRNA and protein expression were assessed by real‐time quantitative PCR and Western blotting in NF‐κB p65‐shRNA cells. (B) NF‐κB p65 mRNA and protein expression were assessed in NF‐κB p65 overexpression cells. (C) Mortalin mRNA and protein expression were assessed in NF‐κB p65‐shRNA cells. (D) Mortalin mRNA and protein expression were assessed in NF‐κB p65 overexpression cells. (E) NF‐κB p65 expression (green) was assessed by immunofluorescence with anti‐NF‐κB p65 primary antibody. DAPI was used to stain nucleus (blue). (F) Mortalin expression (red) was assessed by immunofluorescence with anti‐mortalin primary antibody. Hochest was used to stain nucleus (blue). *P < 0.05, **P < 0.01, ***P < 0.001

NF‐κB p65 mRNA and protein expression were assessed in stable NF‐κB p65 down‐regulated and up‐regulated cell lines using qRT‐PCR and Western blotting. As shown in Figure 2A, the expressions of NF‐κB p65 mRNA and protein in NF‐κB p65 down‐regulated cells (A2780CP NF‐κB p65‐shRNA, A2780S NF‐κB p65‐shRNA) were lower than in control cells (A2780CP‐Ctrl, A2780S‐Ctrl). Conversely, the mRNA and protein expression of NF‐κB p65 in NF‐κB p65 overexpression cells (A2780CP NF‐κB p65‐OV, A2780S NF‐κB p65‐OV) was higher than that in control cells (Figure 2B). Then we detected the expression of mortalin in NF‐κB down‐regulated and up‐regulated cells. Results showed (Figure 2C,D) that mRNA and protein expression of mortalin in A2780CP NF‐κB p65‐shRNA and A2780S NF‐κB p65‐shRNA cells were lower than in control cells and mortalin mRNA and protein expression in A2780CP NF‐κB p65‐OV and A2780S NF‐κB p65‐OV cells were higher than in control cells. Immunofluorescence results (Figure 2E) showed that the fluorescent signal was fainter in the cytoplasm and nucleus of NF‐κB p65‐shRNA cells than in the control cells. At the same time, immunofluorescence results (Figure 2F) also showed that mortalin cytoplasm expression in A2780CP NF‐κB p65‐shRNA and A2780S NF‐κB p65‐shRNA cells was lower than in the control cells. Together, these results indicate that NF‐κB can regulate the expression of mortalin in nucleus.

### 3.3 NF‐κB p65 promotes proliferation and migration of ovarian cancer cells

**Figure 3:**
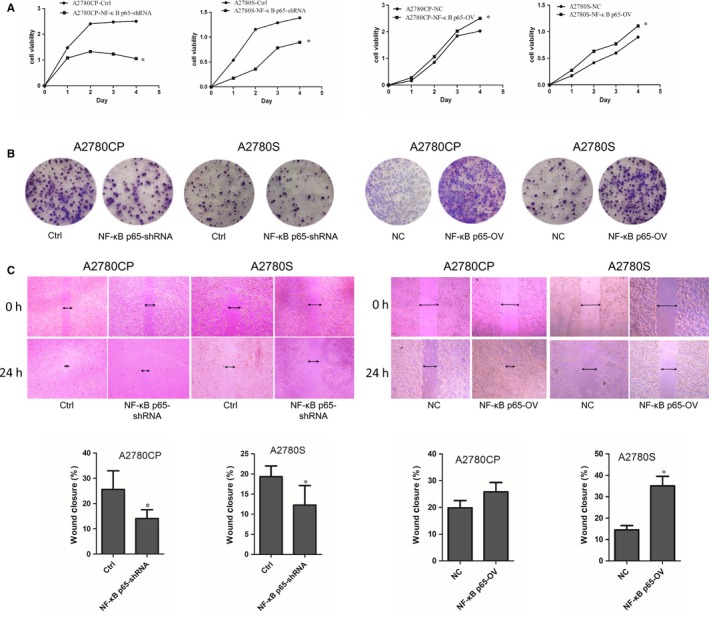
NF‐κB p65 promotes proliferation and migration of ovarian cancer cells. (A) Cell viability was measured using a CCK‐8 assay, which showed that proliferation decreased in NF‐κB p65 down‐regulated cells compared to control group. In contrast, NF‐κB p65 up‐regulated cells exhibited significantly higher growth rates compared to vector controls. (B) Colony formation assays showed that colony size decreased in NF‐κB p65 down‐regulated cells and increased in their NF‐κB p65 up‐regulated counterparts. (C) Wound healing assays showed that NF‐κB p65 overexpression promotes ovarian cancer cells migration. *P < 0.05

The CCK‐8 and colony formation assays were used to investigate the effect of NF‐κB p65 on the proliferation of ovarian cancer cells. The viabilities of A2780CP NF‐κB p65‐shRNA and A2780S NF‐κB p65‐shRNA cells were significantly lower compared with the control groups. In contrast, A2780CP NF‐κB p65‐OV and A2780S NF‐κB p65‐OV cells grew faster than A2780CP‐NC and A2780S‐NC control cells (Figure 3A). As Figure 3B showed that stable NF‐κB p65 down‐regulated cells formed much smaller colonies compared to the control groups. And stable NF‐κB p65 up‐regulated cells formed much larger colonies than control cells. These results indicated that NF‐κB p65 affects the proliferation of ovarian cancer cells.

Wound healing assay was used to assess cell migration. Results (Figure 3C) showed that NF‐κB p65 down‐regulated cells migrated more slowly than control cells. In contrast, NF‐κB p65 up‐regulated cells closed the wound more rapidly compared to controls. These results indicated that NF‐κB affects ovarian cancer cell migration.

### 3.4 NF‐κB p65 promotes proliferation and migration of ovarian cancer cells via regulating mortalin

In order to determine whether NF‐κB p65 influences cell proliferation and migration via mortalin, we transfected the mortalin‐overexpression vector pLVX‐mortalin‐AcGFP into A2780CP NF‐κB p65‐shRNA and A2780CP cells to generate the stable cell lines A2780CP‐NF‐κB p65‐shRNA‐mortalin‐OV and A2780CP‐mortalin‐OV. We then examined the expression of NF‐κB p65 and mortalin using qRT‐PCR and Western blotting.

**Figure 4:**
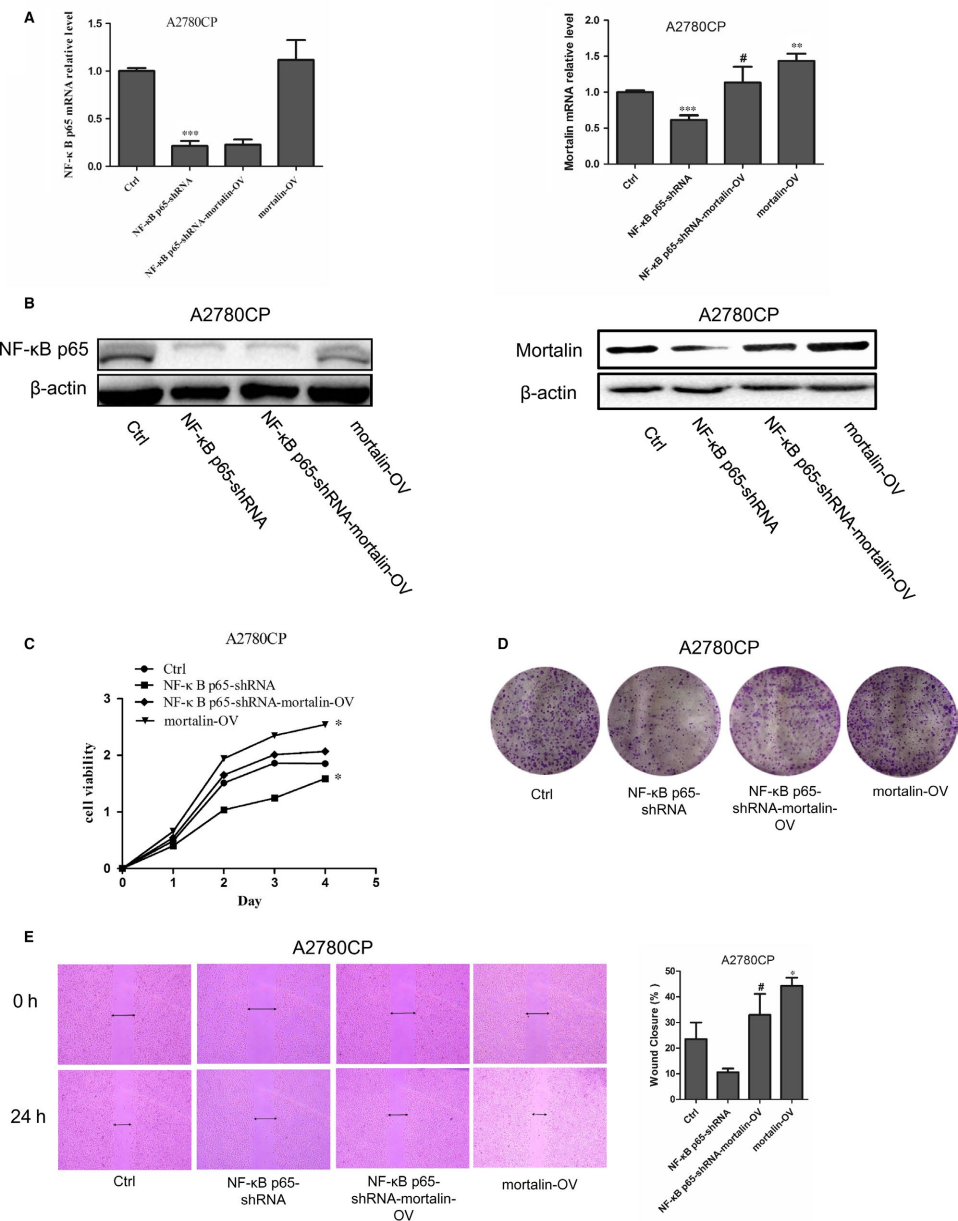
NF‐κB p65 affects the cell viability, proliferation and migration via mortalin in ovarian cancer cells. (A) NF‐κB p65 and mortalin mRNA expression were measured by qRT‐PCR. (B) NF‐κB p65 and mortalin protein expression were measured by Western blotting. (C) Cell viability was measured using a CCK‐8 assay. (D) Colony formation assay presented that the clone formation rate in A2780CP‐NF‐κB p65‐shRNA‐mortalin‐OV group was higher compared to A2780CP‐NF‐κB p65‐shRNA group. (E) Wound healing assay showed that the scratches healing ability of A2780CP‐NF‐κB p65‐shRNA‐mortalin‐OV cells were significantly increased relative to A2780CP‐NF‐κB p65‐shRNA cells. *P < 0.05, **P < 0.01 vs Ctrl, *^#^P* < 0.05 vs A2780CP‐NF‐κB p65‐shRNA

Mortalin mRNA expression in A2780CP‐NF‐κB p65‐shRNA‐mortalin‐OV cells was higher than in A2780CP‐NF‐κB p65‐shRNA cells (Figure 4A); however, no differences were found in A2780CP‐NF‐κB p65‐shRNA‐mortalin‐OV and A2780CP‐mortalin‐OV cells. Consistent with qRT‐PCR results, the mortalin protein expression in A2780CP‐NF‐κB p65‐shRNA‐mortalin‐OV cells was higher than in A2780CP‐NF‐κB p65‐shRNA cells (Figure 4B). These results demonstrate that overexpression of mortalin can reverse the reduction of mortalin expression in A2780CP‐NF‐κB p65‐shRNA cells.

CCK‐8 assays showed that the viability of A2780CP‐NF‐κB p65‐shRNA‐mortalin‐OV cells was slightly higher than that of A2780CP‐NF‐κB p65‐shRNA cells (Figure 4C). Furthermore, the colony formation rate of A2780CP‐NF‐κB p65‐shRNA‐mortalin‐OV cells was higher than A2780CP‐NF‐κB p65‐shRNA cells (Figure 4D). Wound healing assay results (Figure 4E) showed that the migration of A2780CP‐NF‐κB p65‐shRNA‐mortalin‐OV cells was higher than the A2780CP‐NF‐κB p65‐shRNA cells. Taken together, mortalin overexpression can partially rescue the proliferation and migration of ovarian cancer cells reduced by NF‐κB.

## 4 DISCUSSION

Mortalin, a member of the HSP70 family, is widely presented in cells and plays important roles in oxidative stress, regulation of mitochondrial membrane potential, intracellular transport and immune response. It regulates cell proliferation through interacting with a variety of molecules such as P53, GRP94 and VDAC, and participates in a number of molecular pathways.^32^, ^33^, ^34^ Studies show that mortalin expression is elevated in some tumour cells and tissues.^35^ Mortalin overexpression promotes tumorigenesis by inhibiting tumour cells apoptosis and promoting proliferation. In tumour cells, mortalin overexpression can inhibit P53 activity and activate telomerase and hnRNP‐K to promote tumour progression.^36^ In addition, it can inhibit apoptosis by suppressing conformational changes in P53 and BAX.^37^ As an integral part of the ATPase complex, mortalin also plays an important role in maintaining mitochondrial homeostasis, which is critical for the translocation of most mitochondrial membrane and matrix proteins.^38^, ^39^ The energy generated by mitochondria is essential for the process of tumour development.^40^

Our previous studies have found that mortalin can promote cell proliferation, invasion and metastasis by activating Raf/Mek/Erk1/2 cascade signalling pathway and by regulating cell cycle progression in ovarian cancer cells.^11^ However, the mechanism of mortalin expression in the tumour cells remained unclear. We predicted some transcription factors and their binding sites in the mortalin promoter using bioinformatics tools and identified NF‐κB, MZF‐1, LBX‐1 and others as candidates. Li *et al* also showed that NF‐κB may bind to mortalin in human skin keratinocytes (HaCaT) and regulate mortalin expression.^41^ We therefore focused on NF‐κB in this study.

Previous studies have shown that overexpression of mortalin is correlated to the malignancy of ovarian cancer, and that downregulation of mortalin can significantly enhance the sensitivity of cisplatin resistance in ovarian cancer cells and reduce their proliferation and invasion.^42^ Thus, the cisplatin‐resistant human ovarian cancer cell line A2780CP and the cisplatin‐sensitive human ovarian cancer cell line A2780S were selected to study the interaction between NF‐κB p65 and mortalin. We found that NF‐κB p65 expression in A2780CP cells was significantly higher than that in A2780S cells, which was mirrored by mortalin expression. When cells were treated with the NF‐κB p65 inhibitor PDTC, they exhibited different sensitivities to the drug. Low concentrations of PDTC could significantly inhibit NF‐κB and mortalin expression in A2780S cells, whereas higher concentrations were needed in A2780CP cells, suggesting that NF‐κB may be associated with ovarian cancer cell drug resistance.

Other studies have reported NF‐κB overexpression in breast,^25^ lung 43 and bladder cancers 44 and NF‐κB overexpression further promotes the development of tumours. Numerous studies also show that NF‐κB exhibits continuous activation over the course of tumorigenesis. NF‐κB can affect cell cycle progression and promote cell proliferation by directly interacting with cyclin‐D1 and by regulating the expression of various genes such as *BAFFR* and *CD40*.^45^, ^46^, ^47^, ^48^ In addition, NF‐κB can regulate other target genes to inhibit cell apoptosis and promote tumorigenesis. Gong Yang *et al* found that NF‐κB affects the development of ovarian cancer by regulating MAPK signalling pathways and apoptosis.^49^ In low‐grade serous ovarian cancer, NF‐κB can activate proapoptotic signals and act as a tumour suppressor.^50^ Thus, NF‐κB plays an important role in the development and progression of ovarian cancer.

In order to further elucidate the effect of NF‐κB p65 on the proliferation and migration of ovarian cancer cells and on mortalin regulation, we created ovarian cancer stable cell lines with down‐regulated or up‐regulated NF‐κB p65. We then assessed the expression of mortalin and the changes of cell proliferation and migration in these cells. We found that NF‐κB p65 overexpression can increase mortalin expression in ovarian cancer cells and improved the viability, colony formation ability and migration of ovarian cancer cells. At the same time, downregulation of NF‐κB p65 decreased mortalin expression and suppressed ovarian cancer cells proliferation and migration. Subsequently, we transfected a mortalin‐overexpression plasmid into NF‐κB p65 down‐regulated cells (A2780CP NF‐κB p65‐shRNA) and measured the expression of NF‐κB p65 and mortalin. Overexpression of mortalin did not affect the expression of NF‐κB; however, it did rescue the mortalin expression reduced by NF‐κB p65 downregulation. And overexpression of mortalin partly reversed the decreased proliferation and migration in NF‐κB p65 down‐regulated cells. Results of CHIP and luciferase reporter assay indicated that NF‐κB p65 binds to the mortalin promoter at a site with the sequence ‘CGGGGTTTCA’. Taken together, these data suggest that NF‐κB p65 could bind to the promoter region of mortalin and promotes ovarian cancer cells proliferation and migration via mortalin.

It is very interesting, Johnson et al reported that NF‐κB p65 interacted with mitochondrial mortalin under the surveillance of p53.^51^ And we showed that NF‐κB protein transcriptionally increases mortalin expression. Taken together, in the cell nucleus, highly expressed NF‐κB p65 may transcriptionally increase mortalin expression, whereas in mitochondria, the increased mortalin may help with NF‐κB p65 for its mitochondria translocation. This maybe a new mechanism through which NF‐κB can regulate cell growth, migration and metabolism.

Transcription factors and microRNAs are two trans‐acting factor families in eukaryotes, and have different effects on genes. Transcription factors generally regulate gene expression at the transcriptional level, whereas microRNAs act primarily, post‐ transcriptionally. Hillman *et al* found that miR‐200b/c and miR‐217 can bind to mortalin mRNA and decrease its expression in K562 cells.^52^ Our experiments have shown that overexpression of mortalin can partly reverse the proliferation and migration ability of ovarian cancer cells induced by NF‐κB p65 downregulation. Other factors may also regulate mortalin in ovarian cancer cells, and these mechanisms need to be studied further.

In summary, the present study suggests that NF‐κB can bind to the promoter of the molecular chaperone mortalin and promote ovarian cancer cell proliferation and migration via regulating mortalin.

## CONFLICT OF INTEREST

The authors declare that there are no conflicts of interest.

## AUTHOR CONTRIBUTIONS

Shan Li involved in acquisition, analysis, interpretation of data and drafting the manuscript. Mengyuan Lv, Shi Qiu and Jiaqi Meng were involved in acquisition of data. Wen Liu and Ji Zuo revised the manuscript for important intellectual content. Ling Yang supervised the entire study and was involved in design, acquisition, analysis and interpretation of the data and revising the manuscript. All authors reviewed and approved the manuscript.
