# Supplementary material for: NF‐κB p65 promotes ovarian cancer cell proliferation and migration via regulating mortalin
Source: J Cell Mol Med. 2019 Apr 14;23(6):4338–48. doi: 10.1111/jcmm.14325 (PMC6533498; doi:10.1111/jcmm.14325)
Supplement: Supplementary file 1 [file JCMM-23-4338-s001.docx]

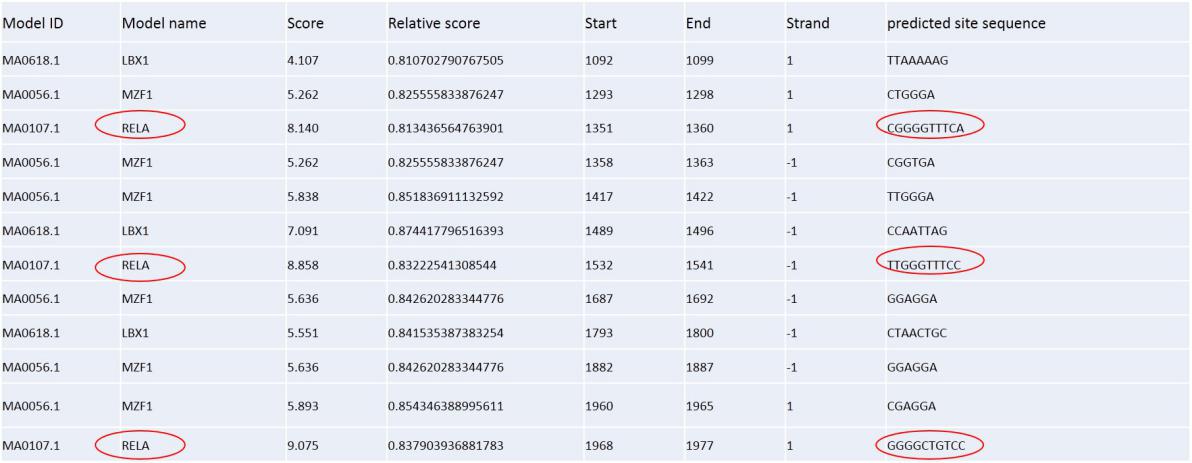


Supplemental table S1: The predict results of mortalin promoter region by

bioinformatic technique

|  | Forward Primer(5'-3') | Reverse Primer(5'-3') |
| --- | --- | --- |
| m1 | TCAGCGGAAGAGCGG | AGGAGTACGAGGCAG |
| m2 | GCCGTTCTCCTGCCTC | GCGAGGTTAGGAGATCGAG |
| m3 | GCCTAATGTTCTGTGTG | TTTGTTGTTTGGTCCCG |
| m4 | CTGGATGTCTGTTGC | GAGGGATATGAAGGC |

Supplemental table S2: Four primer pairs have designed for CHIP-qPCR


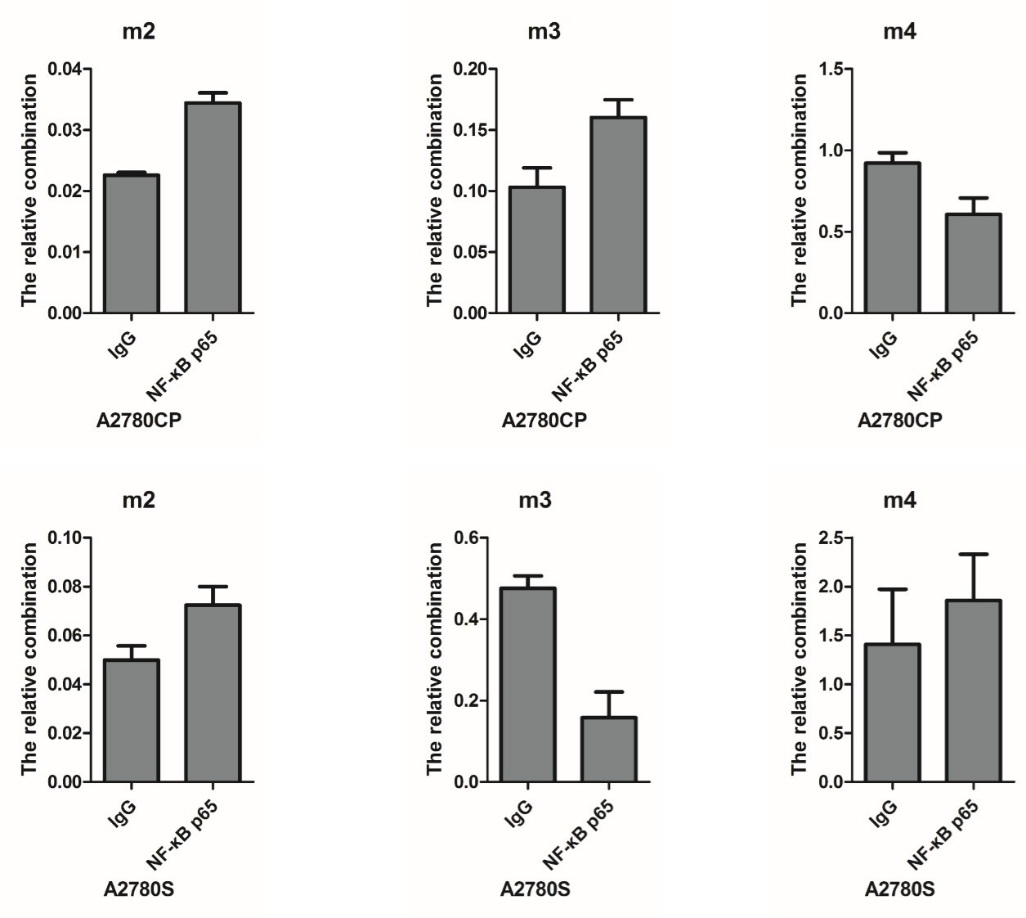


Supplemental FigureS1: The qRT-PCR results of Chromatin immunoprecipitation. There was no significant differences between anti-NF-κB p65 and the control IgG, if the m2, m3, m4 primers were used.
